# Association between gestational diabetes mellitus and risk of breast cancer: a systematic review and meta-analysis

**DOI:** 10.3389/fendo.2025.1621932

**Published:** 2025-07-03

**Authors:** Jing Li, Jinzhu Li, Jie Jin, Ruiqin Zhang, Rong Li, Xian Xu, Yu Wang, Xinghe Hu, Lu Wang, Siyuan Yu

**Affiliations:** ^1^ Department of Geriatric Radiology, The Second Medical Centre & National Clinical Research Centre, Chinese PLA General Hospital, Beijing, China; ^2^ Department of the Sixth Health Care, The Second Medical Centre & National Clinical Research Centre, Chinese PLA General Hospital, Beijing, China; ^3^ Department of Geriatric Emergency, The Second Medical Centre & National Clinical Research Centre, Chinese PLA General Hospital, Beijing, China; ^4^ Department of the First Health Care, The Second Medical Centre & National Clinical Research Centre, Chinese PLA General Hospital, Beijing, China; ^5^ Department of Geriatric Cardiovascular, The Second Medical Centre & National Clinical Research Centre, Chinese PLA General Hospital, Beijing, China

**Keywords:** meta-analysis, gestational diabetes mellitus, breast cancer, systematic review, PRISMA

## Abstract

**Background:**

Gestational diabetes mellitus (GDM), a prevalent metabolic complication during pregnancy, has a global prevalence of approximately 14%. Its onset is closely associated with insulin resistance, insufficient compensatory function of β - cells, and abnormal placental function. Epidemiological studies have indicated that type 2 diabetes is an independent risk factor for breast cancer. However, the association between GDM and the risk of breast cancer remains controversial.

**Objective:**

This systematic review and meta-analysis aim to comprehensively evaluate the association between GDM and the risk of breast cancer and explore its underlying mechanisms.

**Methods:**

This study systematically searched PubMed, Web of Science, Scopus, EMBASE, and the Cochrane Library databases, covering the period from establishing each database until April 14, 2025. Two researchers extracted relevant data and assessed the quality of included studies using the Newcastle-Ottawa Scale. The study evaluated inter-study heterogeneity using the I² statistic. Based on the magnitude of heterogeneity, fixed-effect or random-effect models were employed to calculate the pooled hazard ratio (HR) and its corresponding 95% confidence interval (CI). Additionally, subgroup analyses, sensitivity analyses, funnel plot analyses, and publication bias assessments were performed. All data analyses were conducted using STATA 17 software.

**Results:**

The overall analysis revealed no significant association between GDM and breast cancer risk (HR=1.03, 95%CI: 0.92-1.15). However, subgroup analysis revealed significant regional heterogeneity: within the regional subgroups, North American results showed an association between GDM and a reduced breast cancer risk (HR=0.89, 95%CI: 0.84-0.95), whereas Asian findings suggested an association with an increased risk (HR=1.23, 95%CI: 1.15-1.31). No significant associations were observed in subgroups based on study design (cohort/case-control) or follow-up duration (short-term/long-term). Sensitivity analysis demonstrated robust results, and there was no publication bias in this study.

**Conclusion:**

In summary, there is no significant association between GDM and breast cancer risk overall. However, notable regional heterogeneity exists: in the North American subgroup, GDM is associated with a reduced risk of breast cancer, while in the Asian subgroup, GDM is significantly associated with an increased risk of breast cancer.

**Systematic Review Registration:**

https://www.crd.york.ac.uk/PROSPERO/, identifier CRD420251032589.

## 1 Introduction

Gestational Diabetes Mellitus (GDM) is a glucose metabolism disorder first detected or occurs during pregnancy. It is defined as varying degrees of glucose intolerance during gestation, although the blood glucose level does not meet the diagnostic criteria for overt diabetes mellitus (1–3). As one of the most common metabolic complications during pregnancy, GDM has a global prevalence of approximately 14%. However, due to differences in screening methods, diagnostic criteria, and risk factors such as obesity, advanced maternal age, and family history of diabetes, the incidence rate can fluctuate between 5% and 20% among different populations (4–6). Epidemiological data indicate that the incidence of GDM has been increasing alongside the global rise in obesity and type 2 diabetes mellitus prevalence (4–6). The pathophysiology of GDM is complex and not fully understood. However, it is currently believed that the core mechanisms involve increased insulin resistance and inadequate compensatory function of pancreatic β-cells (2, 3, 7). Physiological insulin resistance during pregnancy is mediated by hormones secreted by the placenta, such as placental lactogen and progesterone. In patients with GDM, genetic susceptibility, environmental factors (e.g., obesity), and pregnancy-related metabolic changes (e.g., increased fat accumulation and secretion of inflammatory and adipokines) exacerbate insulin resistance. At the same time, β-cells cannot fully compensate for these changes (5, 8, 9). Additionally, abnormalities in metabolic reprogramming (such as dysregulation of glycolysis and phosphorylation pathways), oxidative stress, endothelial dysfunction, and epigenetic regulation are thought to contribute to the development and progression of GDM (9–13). The placenta also plays a crucial role in the pathogenesis of GDM, regulating glucose transport between the mother and fetus through glucose transporter proteins (GLUT) and secreting proinflammatory factors that exacerbate insulin resistance (12, 13). Studies have demonstrated that GDM is not only associated with various adverse pregnancy outcomes, such as macrosomia, shoulder dystocia, and preeclampsia but also significantly elevates the long-term risk of metabolic diseases in both mothers and their offspring, including type 2 diabetes mellitus and cardiovascular diseases (3, 5, 14–16). Moreover, recent research has indicated that GDM may influence tumorigenesis through mechanisms such as the insulin-like growth factor-1 pathway and chronic inflammatory state (17, 18) and is linked to the risk of developing cancers like breast cancer.

Breast cancer is the most prevalent malignant tumor among women globally. According to data from the Global Burden of Disease study, there were 2.26 million new breast cancer cases worldwide in 2020, making it the leading cause of cancer-related mortality in women (19, 20). In recent years, multiple studies have conclusively demonstrated that diabetes, including type 2 diabetes (T2DM), represents an independent risk factor for breast cancer (21, 22). This association is likely attributable to promoting tumor cell proliferation by the microenvironment of hyperinsulinemia and hyperglycemia (18, 23).

However, there is a high level of inconsistency in the existing evidence regarding whether GDM independently affects the risk of breast cancer (17, 24–32). For example, Studies by Yong-Moon Mark Park, Oded Fuchs, Sungmin Park, et al. (25–27) suggest a positive correlation between GDM and the risk of breast cancer. Research by Kimberly A Bertrand, Kyu-Tae Han, Maria Hornstrup Christensen, et al. (17, 28, 29) shows no significant association between GDM and breast cancer. Moreover, Camille E. Powe, Dana E. Rollison, S.A.D. Bejaimal et al. (30–32) propose that GDM can reduce the incidence risk of breast cancer. Considering the controversial findings in previous studies and the close relevance of GDM to public health and clinical practice, we conducted a meta-analysis. We aimed to comprehensively evaluate the existing evidence regarding the association between GDM and breast cancer, providing an evidence-based foundation for clarifying the role of GDM in the pathogenesis of breast cancer and formulating risk - stratification management strategies for breast cancer in GDM patients.

## 2 Methods

### 2.1 Registration information

This study was conducted with the requirements of Preferred Reporting Items for Systematic Review and Meta-Analyses guideline (33). And it was registered on the International Prospective Register of Systematic Reviews (ID: CRD420251032589).

### 2.2 Search strategy

We conducted a comprehensive search for original studies on the association between GDM and breast cancer using PubMed, Embase, Scopus, the Cochrane Library, and Web of Science. The search covered the time frame from the inception of each database until April 14, 2025. The search terms consisted of both subject headings and free-text terms. The search strategy employed for PubMed was as follows: (((“Diabetes, Gestational”[Mesh]) OR (((Gestational Diabetes Mellitus) OR (GDM)) OR (Diabetes, Pregnancy-Induced))) AND ((“Neoplasms”[Mesh]) OR (((Cancer*) OR (Tumor*)) OR (Carcinoma*)))) AND (Breast OR mammary gland). This strategy was adapted for use in the other databases, with terminology adjustments made according to each database’s specific syntax and indexing system. Meanwhile, manual searches were carried out based on the reference lists of relevant studies.

### 2.3 Eligibility criteria

Eligible studies must meet the following criteria:

(1) The study design must be a cohort or case-control study.

(2) The study should focus on the association between GDM and breast cancer risk.

(3) The study should report odds ratios (OR), relative risks (RR), or hazard ratios (HR) with their corresponding 95% confidence intervals (CI) or provide sufficient data to calculate the effect size between GDM and breast cancer.

(4) The article must be published in English.

### 2.4 Study selection

The search results from various databases were imported into Endnote X9 software for deduplication and literature management. To ensure data accuracy and objectivity, two independent reviewers (JL and JZL) screened the titles and abstracts of the retrieved literature based on pre-set inclusion criteria. Full texts were obtained and further screened for studies that passed the initial screening to determine the final included studies. During the screening process, any disagreements between the two reviewers were resolved through discussion to reach a consensus; if necessary, a third reviewer (JJ) would participate in the discussion and provide an arbitration opinion.

### 2.5 Data extraction

This study strictly adhered to the PRISMA statement for data extraction to ensure the systematic approach of the research methodology. Two reviewers (JL and JZL) independently extracted data using a predefined data extraction form, while a third author (JJ) cross-checked the accuracy of the results. The extracted data included Author (year), Country, Study design, Age (GDM/non-GDM), Sample size, RR (95% CI), OR (95% CI), HR (95% CI), Follow-up time (years), Quality (scores), and Adjustment factors.

### 2.6 Quality assessment

This study evaluated the quality of the included studies using The Newcastle-Ottawa Quality Assessment Scale. The Newcastle-Ottawa Quality Assessment Scale is a tool specifically designed to assess the quality of both cohort and case-control studies, enabling the evaluation of the quality of each study (34). It consists of 8 items organized into three domains: selection of the study groups, comparability of the groups, and assessment of exposure/outcome. The maximum score on this scale is 9 points. Studies scoring less than 4 points are considered low quality, those scoring between 4 and 6 points are of moderate quality, and those scoring between 7 and 9 points are deemed high quality (34).

### 2.7 Data synthesis and analysis

To evaluate the association between GDM and breast cancer, we equated all RR and OR to HR (35) and then conducted a meta-analysis of HR and its 95% CI. We used the Q test to assess heterogeneity among studies, with a significance level set at P = 0.1. Subsequently, we determined the degree of heterogeneity based on the I² statistic: if I² < 50%, indicating non-significant heterogeneity, we applied a fixed-effects model; if I² ≥ 50%, suggesting significant statistical heterogeneity, we chose a random-effects model (36). Additional analyses were conducted, including a sensitivity analysis using the leave-one-out method (37) and an assessment of publication bias by observing funnel plot symmetry and calculating Begg’s and Egger’s test values (38, 39). Data were processed using Stata 17.0 statistical software, with P < 0.05 indicating statistical significance.

## 3 Results

### 3.1 Compliance with the registered protocol

There were no inconsistencies with the pre-registration protocol.

### 3.2 Study selection

**Figure 1:**
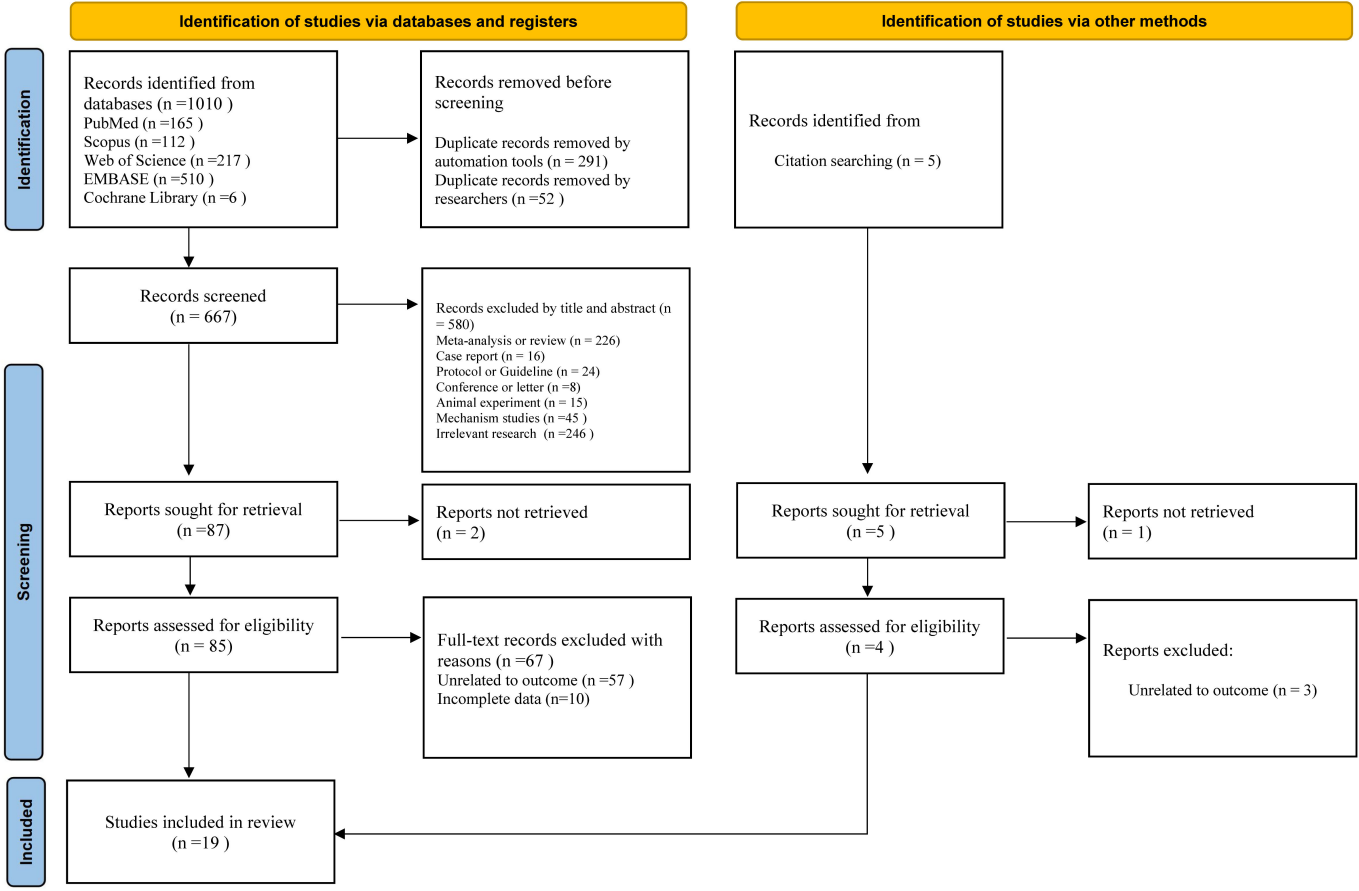
PRISMA flow diagram depicting the process of study selection for meta-analysis.

**Figure 1** illustrates the selection process and reasons for exclusion in this study. We retrieved 1010 articles from five databases: PubMed, Embase, Scopus, Cochrane Library, and Web of Science. In this study, 343 duplicate articles were removed using the automatic tools of Endnote X9 and manual efforts. Subsequently, 580 studies were excluded based on their titles and abstracts, leaving 87 articles for further evaluation. Two studies were excluded due to the inability to obtain the full text, and the remaining 85 studies progressed to the full-text evaluation stage. After full-text evaluation, 67 studies were excluded because their outcome measures were not relevant to the theme of this study, or complete target data could not be obtained. Ultimately, 18 studies met the inclusion criteria and were included. A related citation tracking search and supplementation were also conducted (5 studies). After applying the inclusion and exclusion criteria, one additional study was included. Finally, 19 studies (17, 25–32, 40–49) were obtained for meta-analysis.

### 3.3 Study characteristics

This meta-analysis included 19 studies, consisting of 14 cohort studies and five case-control studies, spanning multiple countries and regions such as Canada, the United States, Israel, South Korea, Taiwan (China), and Denmark. The sample size of the included studies ranged from a minimum of 630 cases to a maximum of 990,572 cases, with a total sample size exceeding 3 million. These studies employed hazard ratios (HR), odds ratios (OR), or relative risks (RR) to evaluate the strength of the association between GDM and breast cancer. Four studies indicated a reduced risk of breast cancer with GDM, five studies showed an increased risk, and ten studies found no significant association. Except for one study rated as moderate quality (5 scores), all other studies were of high quality (≥7 scores). All studies adjusted for confounding factors, covering multiple dimensions such as age, parity, BMI, pregnancy-related factors, long-term lifestyle, and disease history. More information about the main results of each study is presented in **Table 1**.

**Table 1:** Characteristics of individual studies included in the meta-analysis.

Author (year)	Country	Study design	Age (GDM/ non-GDM)	Sample size	RR (95% CI)	OR (95% CI)	HR (95% CI)	Follow-up time (years)	Quality (scores)	Adjustment factors
Gurjot Gill MD,2024	Canada	Cohort study	33(IQR 33-37)	297,771			0.90 (0.82, 0.98)	8 (IQR 4-13)	High (7)	Adjusted 1
Kimberly A Bertrand,2021	USA and Sweden	Cohort study	38(IQR 20-54)	257,290			0.90 (0.78, 1.03)	16 (IQR 0.1-24)	High (7)	Adjusted 2
Tal Sella,2011 (43)	Israel	Cohort study	32.74 (SD 5.51)/ 30.59 (SD 5.51)	185,315			0.87 (0.63, 1.20)	5.19 (SD 3.9)	High (8)	Adjusted 3
Oded Fuchs,2017 (26)	Israel	Cohort study	31.8 (SD 5.9)/ 28.1 (SD 5.9)	104,715		2.0 (1.595,2.51)		11.2 (Average)	High (7)	Adjusted 4
Kyu-Tae Han,2018 (28)	South Korea	Cohort study	28.25 (SD 3.28)/ 27.28 (SD 3.02)	102,900			1.15(0.831, 1.581)	10	High (8)	Adjusted 5
YunShing Peng,2019 (46)	Taiwan, China	Cohort study	31.61 (SD4.54)/ 28.83 (SD4.89)	990,572			1.234 (1.093, 1.393)	6.84 (SD 3.05)	High (7)	Adjusted 6
Yong-Moon Mark Park,2017 (25)	USA	Cohort study	GDM-1T* 51.6 (SD 8.2); GDM-2T 51.2 (SD 7.9)/ 56.1 (SD 9.0)	39,198			1.68 (1.15, 2.44)	7.4 (Average)	High (8)	Adjusted 7
S.A.D.Bejaimal,2015 (32)	Canada	Cohort study	32 (IQR 28–35)	149,049			0.86 (0.75, 0.98)	8 (IQR 5-12)	High (7)	Adjusted 8
Kimberly A. Bertrand,2020 (28)	USA	Cohort study	36.5/ 41.0	41,767			0.98 (0.77, 1.25)	22 (Average)	High (8)	Adjusted 9
Theodore M. Brasky,2013 (44)	USA	Case-control study	35-79	2818	0.79 (0.48, 1.30)				High (7)	Adjusted 10
Camille E. Powe,2017 (30)	USA	Cohort study	33.8 (SD 4.4)/ 35.0 (SD4.7)	86,972			0.68 (0.55, 0.84)	22 (Average)	High (7)	Adjusted 11
Romina Pace,2020 (47)	Canada	Cohort study	Not available	68,588			0.93 (0.80, 1.09)	13.1 (SD 5.2)	High (7)	Adjusted 12
M. C. Perrin,2008 (41)	Israel	Cohort study	Not available	40,898			1.5 (1.0, 2.1)	34 (Median)	High (8)	Adjusted 13
Maria Hornstrup Christensen,2024 (17)	Denmark	Cohort study	28(IQR 25-32)/ 28(IQR25 - 31)	708,121			0.96 (0.83, 1.12)	11.9 (IQR 0-21.9)	High (7)	Adjusted 14
Arash Ardalan,2016 (45)	USA	Case-control study	Not available	630		1.62 (0.30, 8.68)			High (8)	Adjusted 15
Maureen Sanderson,2010 (42)	USA	Case-control study	33-79	1669		0.26 (0.03, 2.31)			High (7)	Adjusted 16
Dana E. Rollison,2008 (31)	USA	Case-control study	57/ 55.5	2324		0.70 (0.51, 0.96)			High (8)	Adjusted 17
Rebecca Troisi,1998 (40)	USA	Case-control study	22-44	2405	1.1 (0.83, 1.5)				Medium (5)	Adjusted 18
Sungmin Park,2022 (27)	South Korea	Cohort study	Not available	235,872			1.15 (1.05, 1.27)	12	High (7)	Adjusted 19

GDM, Gestational Diabetes Mellitus; RR, Relative Risk; OR, Odds Ratio; HR, Hazard Ratio; CI, Confidence Interval; IQR, Interquartile Range; SD, Standard Deviation.

GDM-T*: Number of times having GDM.

Adjusted 1 Age, parity, year of delivery, neighbourhood income quintile, urban vs rural residence, recent immigration status, surname - based ethnicity, number of core primary care visits in 3 years before delivery, endocrinologist visits in follow - up period.

Adjusted 2 Age, race/ethnicity, attained education, parity, age at first birth, age at most recent birth, and young adult body mass index (BMI).

Adjusted 3 Age, socioeconomic level, smoking status, BMI, parity, number of general practitioner visits 2 years prior to the index date.

Adjusted 4 Fertility treatment, maternal age, and parity.

Adjusted 5 Maternal age, smoking, BMI before pregnancy and FBG.

Adjusted 6 Age, hypertension, dyslipidemia, liver disease, infertility and kidney disease.

Adjusted 7 Birth cohort, race or ethnicity , educational attainment , age at first birth, age at menarche, relative weight at age 10, BMI at 30–39 years old and physical activity in their childhood and teens.

Adjusted 8 Income, and number of physician visits in the 3 years before the index date.

Adjusted 9 age, questionnaire cycle, body mass index at age 18, recent body mass index, parity, menarche, age at first birth, oral contraceptive duration, and family history of breast cancer.

Adjusted 10 Age, education, history of benign breast disease, family history of breast cancer, age at first pregnancy, number of pregnancies, menopausal status, and age at menopause (among postmenopausal women)

Adjusted 11 BMI at age 18, weight gain since age 18, height, total physical activity, alcohol intake, age at menarche, birth index, total breastfeeding, menopausal status, hormone therapy use, family history of breast cancer in mother or sister, personal history of benign breast disease, White race/ethnicity, mammography within the past 2 years. Additionally, supplemental models adjusted for self-reported pregnancy-associated hypertension and use of diabetes therapies.

Adjusted 12 Gestational hypertension, preterm delivery, infant size, parity, prior comorbidity, material deprivation index, and ethnicity.

Adjusted 13 Age, birth order at the first observed birth, social class, ethnic origin, education, and immigration status.

Adjusted 14 Age at index pregnancy, parity, preexisting hypertension, preexisting comorbidity, ethnicity, marital status, income, education, occupation, and calendar year of delivery.

Adjusted 15 Maternal age at delivery, race/ethnicity, level of education, birth weight, parity, gestational age, weight gain during pregnancy, smoking habit, drinking habit, induction of labor, gestational hypertension.

Adjusted16 Menopausal status and mammography screening.

Adjusted 17 Age, body mass index at age 15 years, and number of full-term pregnancies.

Adjusted 18 Study site, age (as a continuous variable), race, number of births, and other breast cancer risk factors associated with the evaluated pregnancy characteristics (such as parity, age at first birth, years of oral contraceptive use, etc.).

Adjusted 19 Age at diagnosis of breast cancer, age at first delivery, age at last delivery, number of deliveries, interval between deliveries, and treatment methods (endocrine therapy, chemotherapy, targeted therapy).

### 3.4 Overall meta-analysis

**Figure 2:**
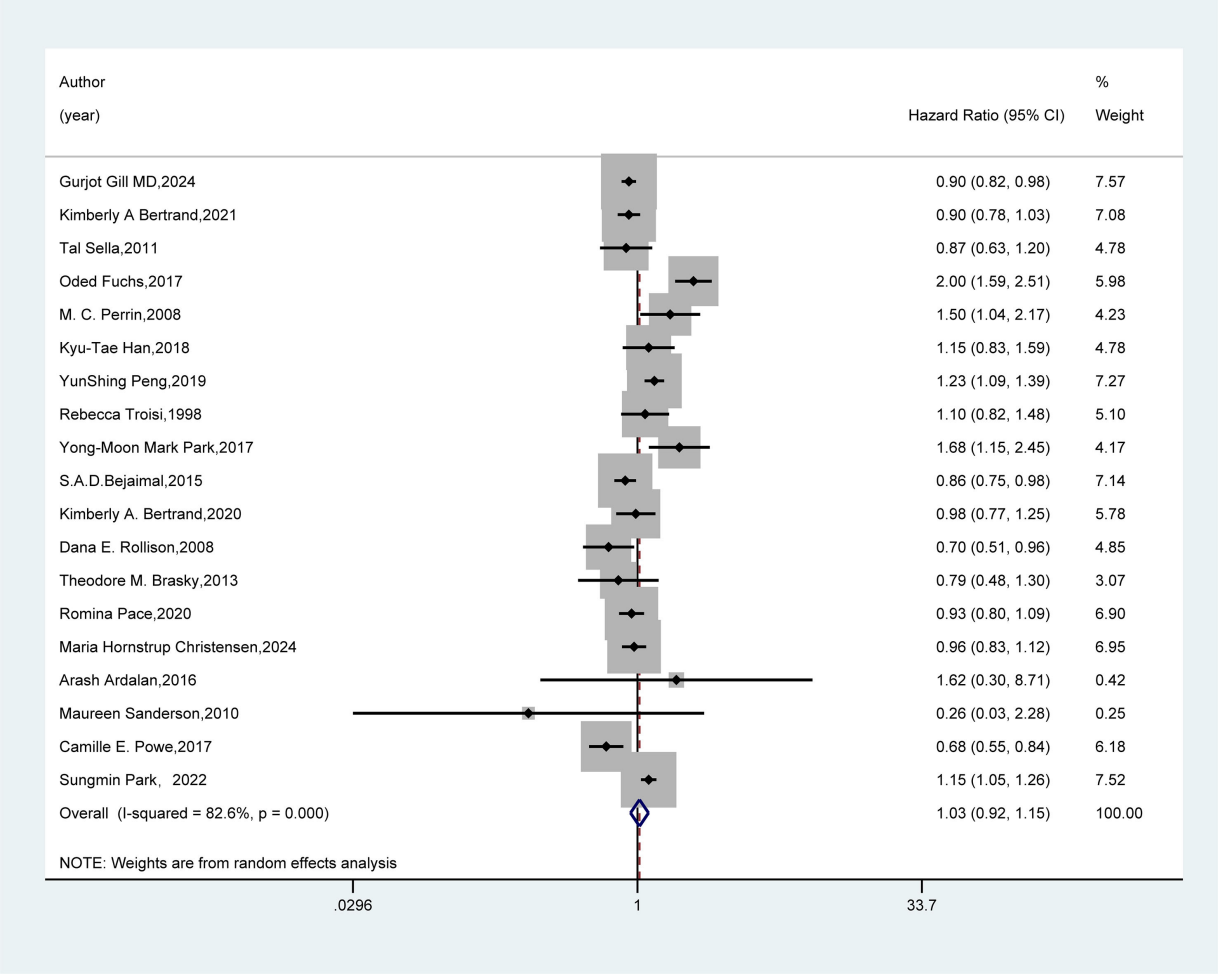
Forest plot of random-effects meta-analysis of the association between GDM and breast cancer risk. A random-effects model was used for the Meta-analysis to evaluate the hazard ratio (HR) and 95% confidence interval (CI) of the association between gestational diabetes mellitus (GDM) and breast cancer. A rectangle represented the HR value of each study, and the weight was marked on the right side (for example, the weight of Gurjot Gill MD, 2024 was 7.57%). The horizontal line indicated the range of the 95% CI. The diamond at the bottom represented the pooled effect size: HR = 1.03 (95% CI: 0.92 - 1.15). The heterogeneity test showed that 1-square (I²) = 82.6%, suggesting a high degree of heterogeneity among the studies. The weights were determined by the random-effects model, reflecting the contribution of each study to the pooled results.

We conducted a systematic review and meta-analysis to evaluate the association between GDM and breast cancer risk by including 19 cohort or case-control studies. The results (**Figure 2**) showed no significant association between GDM and the risk of developing breast cancer (HR=1.03, 95%CI: 0.92-1.15).

### 3.5 Subgroup analyses

**Figure 3:**
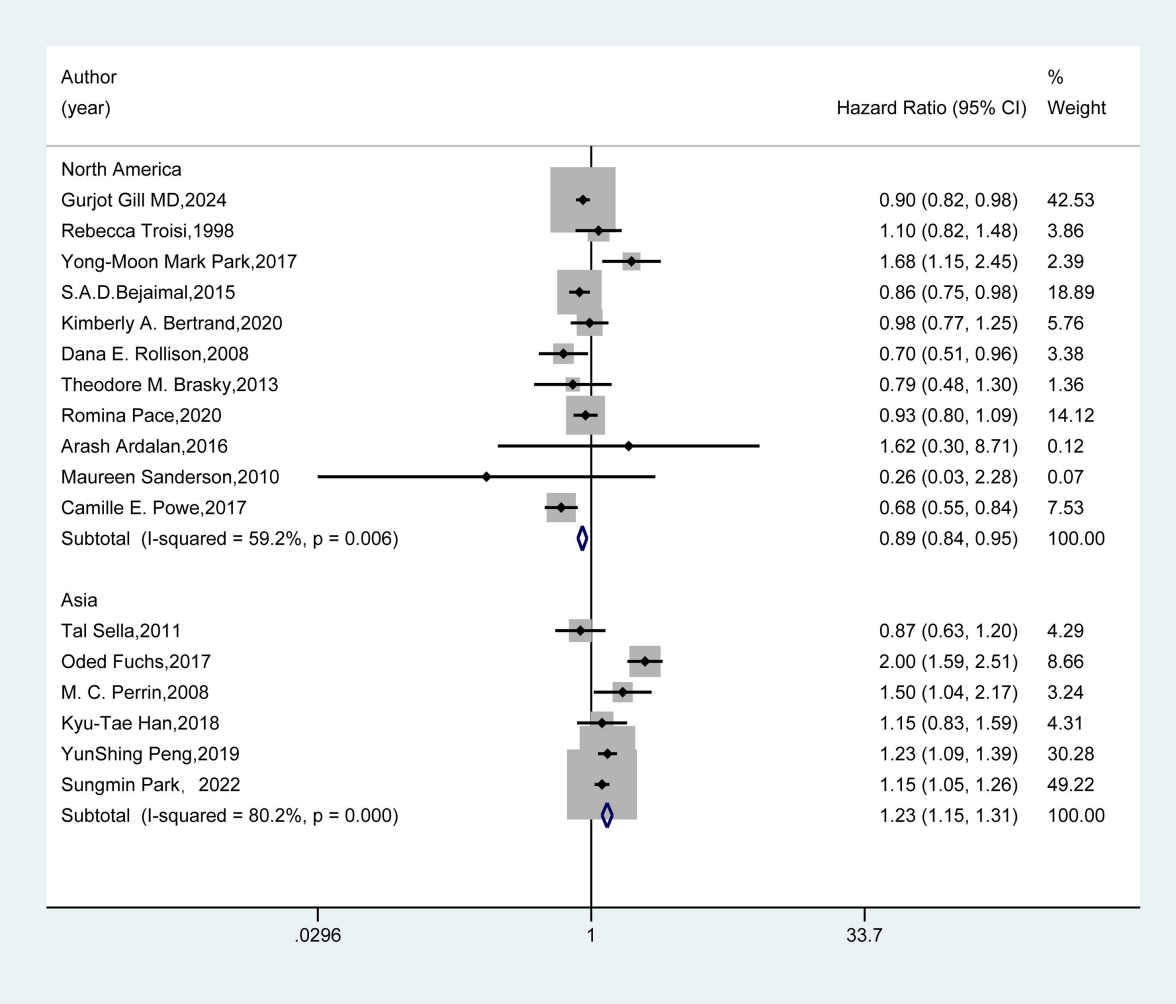
Subgroup analysis - region. A random-effects model was employed for the Meta-analysis to assess the hazard ratio (HR) and 95% confidence interval (CI) of the association between gestational diabetes mellitus (GDM) and breast cancer in different regional subgroups. The HR value of each study was presented as a rectangle, with the weight marked on the right side. The horizontal line represented the range of the 95% CI. The diamond at the bottom represented the pooled effect size, and I² represented the degree of heterogeneity among the studies. The weights were determined by the random-effects model, reflecting the contribution of each study to the pooled results.

We conducted subgroup analyses based on the included studies’ region, study design type, and follow-up duration. In the regional subgroup (**Figure 3**), results from 11 studies in North America showed that GDM could reduce the risk of breast cancer (HR=0.89, 95%CI: 0.84-0.95). Conversely, findings from six studies in Asia indicated that GDM increased the risk of breast cancer (HR=1.23, 95%CI: 1.15-1.31).

**Figure 4:**
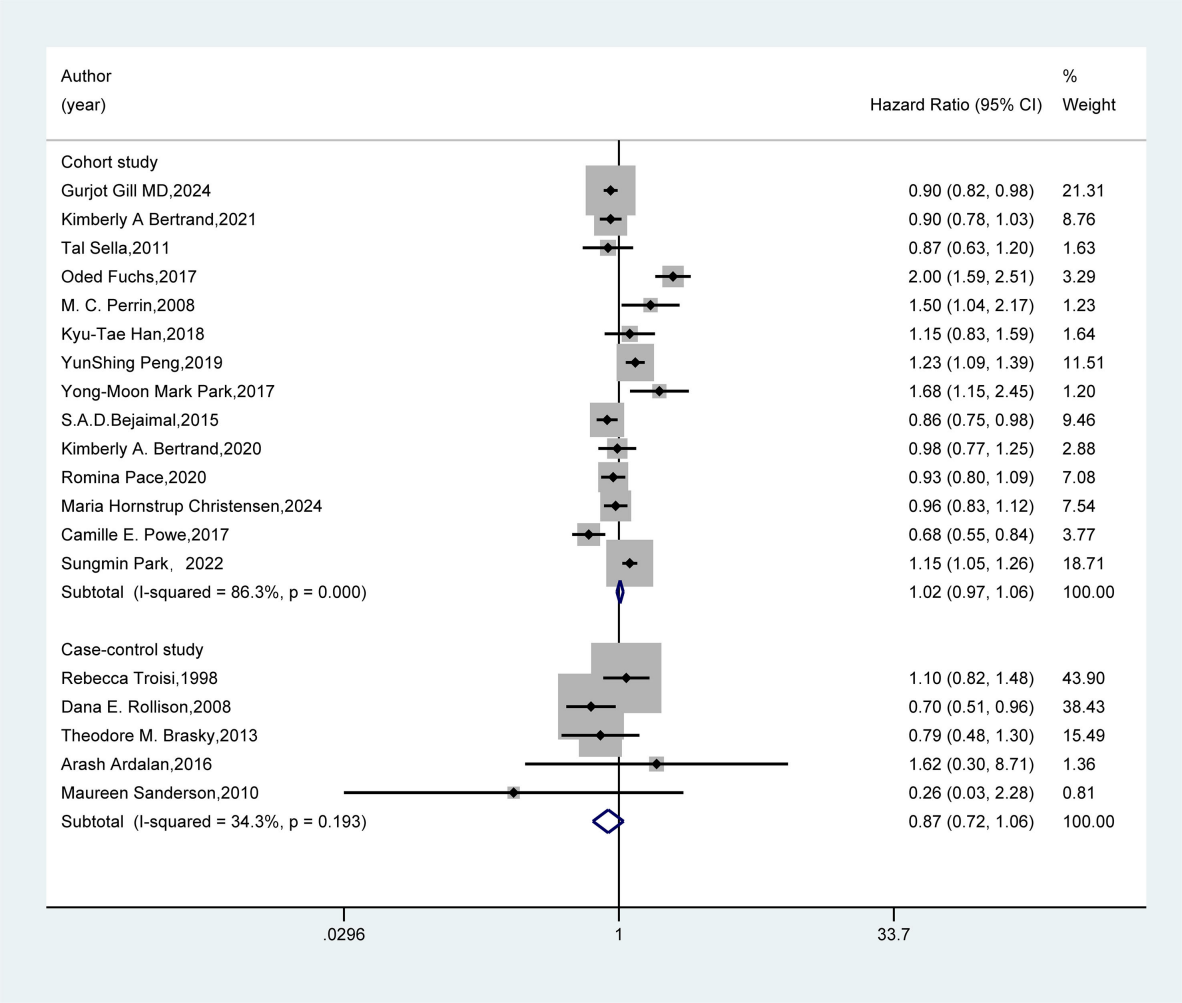
Subgroup analysis - study design. A random-effects model was used for the Meta-analysis to evaluate the hazard ratio (HR) and 95% confidence interval (CI) of the association between gestational diabetes mellitus (GDM) and breast cancer in the subgroup of cohort studies, while a fixed-effects model was employed for the evaluation of case-control studies. A rectangle represented the HR value of each study, and the weight was marked on the right side. The horizontal line indicated the range of the 95% CI. The diamond at the bottom represented the pooled effect size, and I² represented the degree of heterogeneity among the studies. The weights were determined by the random-effects model, reflecting the contribution of each study to the pooled results. If I² < 50%, it indicates non-significant heterogeneity, and a fixed-effects model should be used. If I² ≥ 50%, significant statistical heterogeneity is considered to exist, and a random-effects model should be selected.

In the study design subgroup (**Figure 4**), the pooled analysis of 14 cohort studies and five case-control studies both demonstrated no association between GDM and the risk of breast cancer (HR=1.02, 95%CI: 0.97-1.06; HR=0.87, 95%CI: 0.72-1.06).

**Figure 5:**
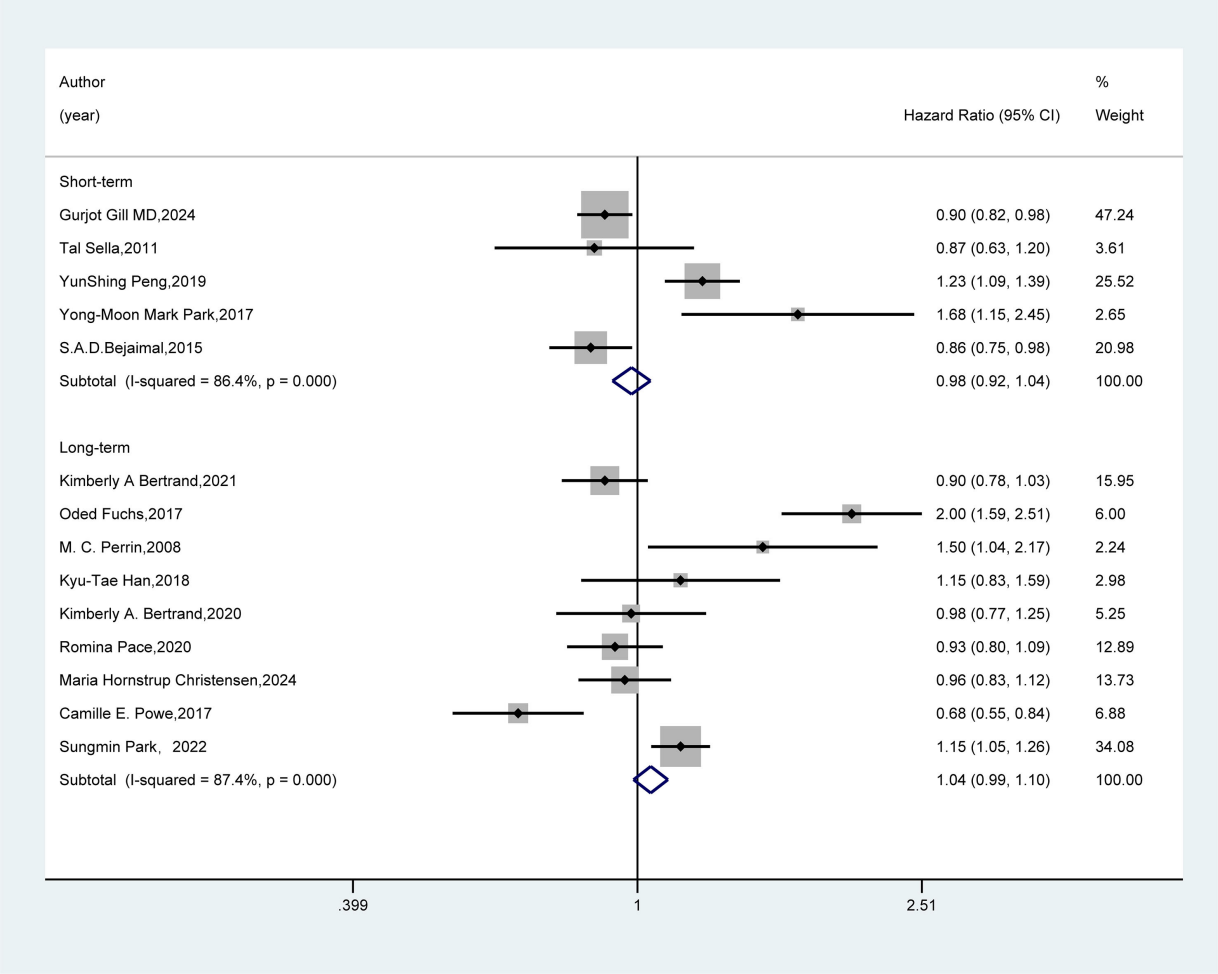
Subgroup analysis - follow-up duration. Meta-analysis was performed using a random-effects model to assess the hazard ratio (HR) and 95% confidence interval (CI) of the association between GDM and breast cancer in different follow-up duration subgroups. A rectangle represented the HR value of each study, and the weight was marked on the right side. The horizontal line indicated the range of the 95% CI. The diamond at the bottom represented the pooled effect size, and I² represented the degree of heterogeneity among the studies. The weights were determined by the random-effects model, reflecting the contribution of each study to the pooled results.

In the follow-up duration subgroup (**Figure 5**), the combined results from five studies with short-term follow-up and nine studies with long-term follow-up revealed no significant association between GDM and the risk of breast cancer (HR=0.98, 95%CI: 0.92-1.04; HR=1.04, 95%CI: 0.99-1.10).

### 3.6 Sensitivity analysis

**Figure 6:**
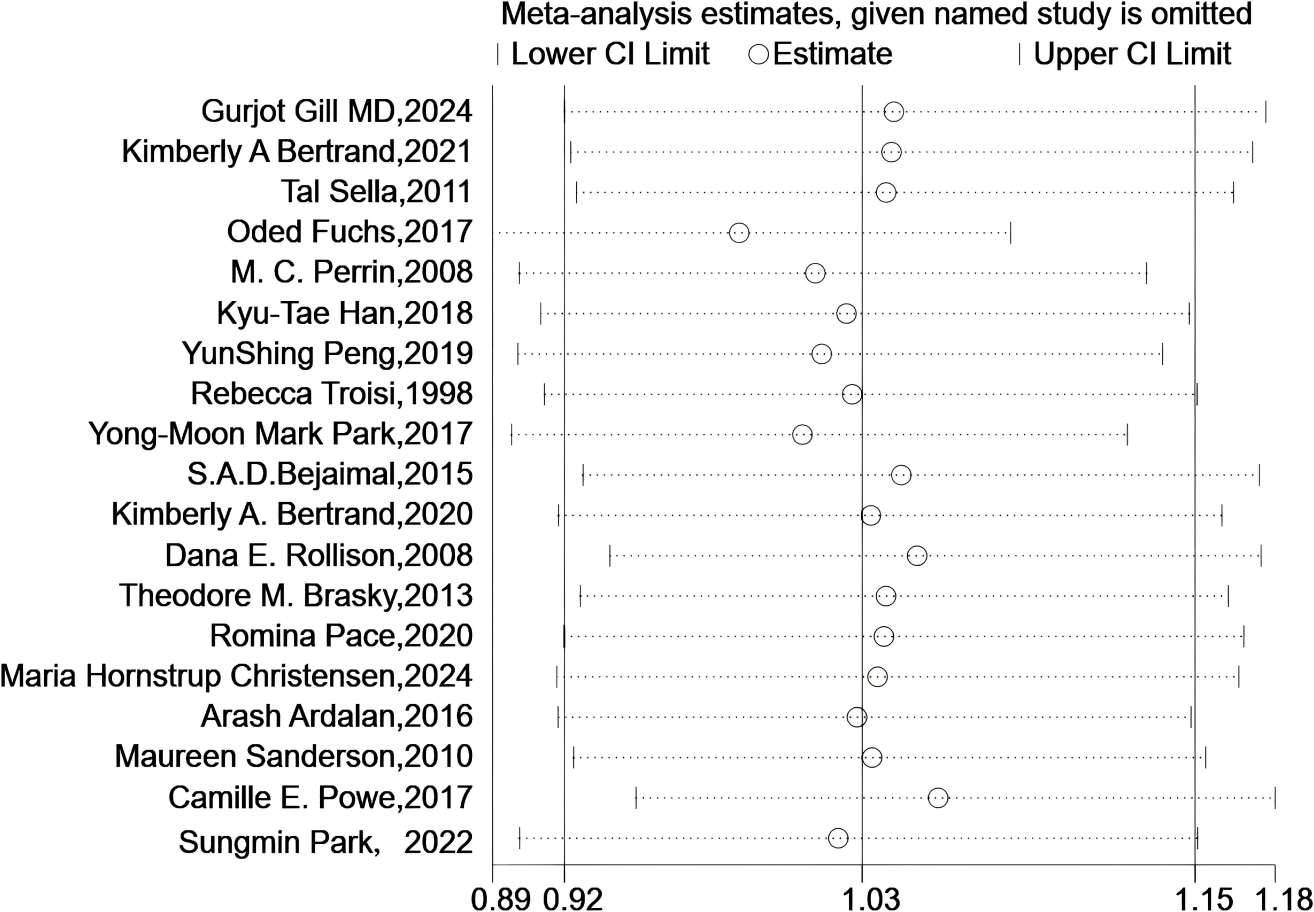
Sensitivity analyses. Sensitivity analysis plot shows meta-analysis estimates when each named study is omitted. The circles represent the effect size estimates, and the horizontal lines denote the 95% confidence intervals (lower and upper limits). Each row corresponds to a study excluded one - by - one, illustrating how the overall meta-analysis result changes with the removal of individual studies.

As shown in **Figure 6**, the sensitivity analysis revealed that the pooled results remained robust after excluding any individual study.

### 3.7 Publication bias

**Figure 7:**
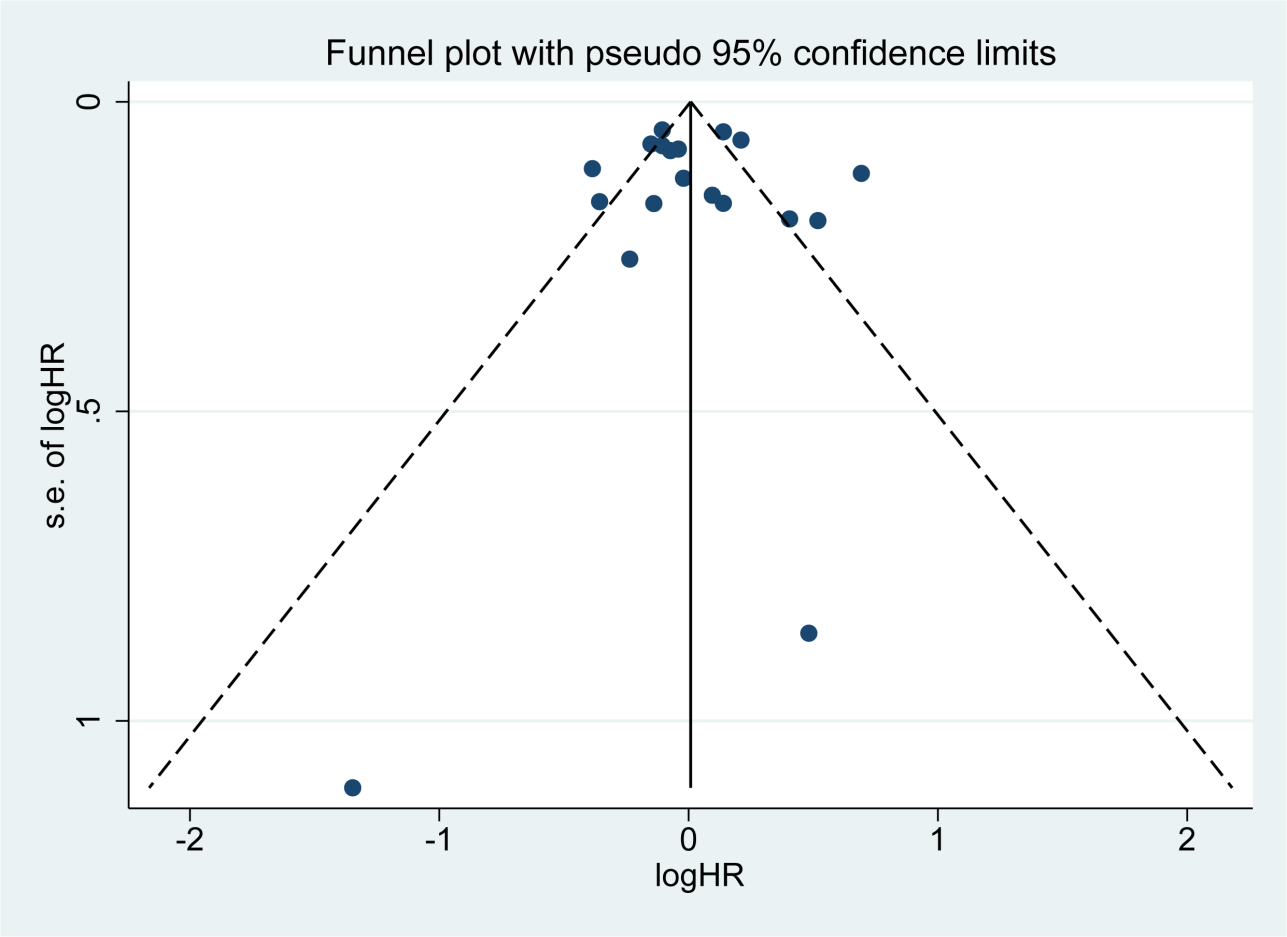
Funnel plot. A small black dot represents a single study. If all the small black dots are symmetrically distributed, it can be considered that there is no significant publication bias in the results of the meta-analysis. Conversely, if they are not symmetrically distributed, significant publication bias exists.

The funnel plot (**Figure 7**), Begg’s test (Z = 0.63, P = 0.529), and Egger’s test (T = 0.21, P = 0.835) (**Supplementary Figure S1**) provided additional evidence supporting the absence of publication bias in our meta-analysis summary results.

## 4 Discussion

GDM is abnormal glucose metabolism that first appears or is diagnosed during pregnancy, with its pathophysiological characteristics closely linked to insulin resistance (17, 50). In recent years, the incidence of GDM has risen significantly alongside the global epidemic of obesity and T2DM (51). Numerous studies have demonstrated an association between T2DM and an increased risk of breast cancer (21, 50). However, the relationship between GDM and breast cancer remains controversial (26, 45, 49). To clarify this association, we conducted a meta-analysis synthesizing existing epidemiological evidence from 19 studies. Our findings indicate that GDM is not associated with the risk of breast cancer. However, subgroup analysis revealed a regional variation in this association: in the North America subgroup, GDM was found to decrease the risk of breast cancer, while in the Asia subgroup, it was associated with an increased risk. This disparity suggests that regional distribution may be a crucial factor influencing the association between GDM and breast cancer risk.

### 4.1 Potential mechanisms of GDM in the development and progression of breast cancer

Currently, there is controversy regarding mechanistic studies on the relationship between GDM and breast cancer risk. From a metabolic perspective, the unique state of insulin resistance in women with GDM may affect the growth conditions of tumor cells by altering the microenvironment of breast tissue (17). High estrogen and progesterone levels during pregnancy can induce breast cell differentiation, which may provide a protective effect, reducing the sensitivity of breast epithelial cells to carcinogenic factors (2). Additionally, women with GDM often require strict glycemic control and lifestyle interventions, such as dietary adjustments and moderate exercise. These measures indirectly influence breast cancer risk by improving the overall metabolic state (52). It is worth noting that breastfeeding after GDM may play a significant role, as it promotes terminal differentiation of breast epithelial cells and extends the recovery period of hormone exposure. This biological change may have a long-term protective effect on breast tissue (53, 54). Some studies have also found that changes in specific metabolites associated with GDM (such as adiponectin) can affect tumorigenesis by regulating inflammatory responses and cell proliferation pathways (55, 56).

However, there are also studies suggesting that GDM may increase the risk of breast cancer. Firstly, patients with GDM exhibit significant insulin resistance and hyperinsulinemia. Insulin and its growth factors (such as IGF-1) can promote the proliferation of breast epithelial cells and inhibit apoptosis by activating signaling pathways like PI3K/Akt and MAPK, thereby increasing the risk of carcinogenesis (17, 57). For GDM patients carrying breast cancer genetic susceptibility genes (such as germline mutations in BRCA1/2), hyperinsulinemia, and chronic inflammation may further impair the DNA damage repair capacity through the PI3K/Akt pathway (58, 59). Secondly, the chronic hyperglycemic state associated with GDM can lead to oxidative stress and the accumulation of advanced glycation end products (AGEs), which can induce DNA damage and genomic instability (60, 61). Furthermore, the abnormal secretion of inflammatory factors (such as IL-6 and TNF-α) from adipose tissue in women with GDM can create a tumor-promoting microenvironment. At the same time, elevated estrogen and progesterone levels during pregnancy may synergistically promote the development of breast cancer through hormonal receptor pathways (21, 62). It is important to note that some patients may develop type 2 diabetes mellitus (T2DM) after GDM, and the accompanying metabolic syndrome (such as obesity and dyslipidemia) may further exacerbate the risk of breast cancer by altering adipose factors (such as imbalances in the leptin/adiponectin ratio) (57, 63). These conflicting mechanisms suggest that the impact of GDM on breast cancer may involve complex metabolic memory effects and individual differences (60, 64).

### 4.2 Possible mechanisms for the differential results in the regional subgroups

The subgroup analysis in this study revealed a trend toward reduced breast cancer risk in women with GDM in North America. In contrast, a significant positive association between GDM and increased breast cancer risk was observed in Asia. These results may be attributed to variations in diagnostic criteria, accessibility to healthcare services (including medical interventions), regional lifestyle, body mass index (BMI) cutoff values, breastfeeding practices, and genetic factors.

In North America, the potential association between GDM and reduced breast cancer risk may be attributed to the following factors: Firstly, regarding diagnostic criteria and healthcare services, North America adopts the International Association of Diabetes and Pregnancy Study Groups (IADPSG) diagnostic criteria for GDM, enabling accurate patient identification and targeted management (65). Concurrently, the monitoring and intervention for postpartum metabolic abnormalities (e.g., insulin resistance, obesity) in GDM patients are more comprehensive and systematic. Through lifestyle modifications such as dietary control and physical activity, the long-term risk of metabolic disorders is mitigated, thereby reducing potential breast cancer-promoting factors (17). Furthermore, the high accessibility of healthcare services in North America ensures that patients receive timely professional advice and treatment, which facilitates better disease management (17). Secondly, regarding obesity, screening, and breastfeeding, The high obesity rate among North American women, combined with higher BMI thresholds, leads to increased clinical attention toward a larger cohort of obese females. As a marker of metabolic aberration, GDM prompts earlier initiation of breast cancer screening (e.g., mammography), enabling early lesion detection and statistically manifesting as “risk reduction” (21, 57). Additionally, the relatively prevalent breastfeeding practice in North America promotes terminal differentiation of mammary epithelial cells. It prolongs the recovery period from hormonal exposure, conferring long-term protective effects on breast tissue and reducing breast cancer risk. Thirdly, from a genetic perspective, Genetic polymorphisms associated with GDM (e.g., TCF7L2, IRS1) in North American populations (particularly those of European ancestry) (66) may intersect with breast cancer protective pathways (e.g., estrogen metabolism), counteracting the carcinogenic effects of hyperglycemia (2, 17).

In Asian populations, the potential association between GDM and increased breast cancer risk may be explained by the following mechanisms: First, regarding diagnostic criteria and healthcare services, the diagnostic thresholds for GDM in Asian populations are relatively lenient (67, 68), potentially including more mild hyperglycemia cases. The metabolic abnormalities in these cases often receive insufficient intervention, which may lead to epigenetic carcinogenic effects (69, 70). Additionally, the accessibility of healthcare services is suboptimal in some Asian regions, making it difficult for GDM patients to obtain timely and comprehensive medical care, thereby compromising disease management (17). Second, due to the persistent impact of metabolic dysfunction, Asian GDM patients exhibit higher rates of progression to type 2 diabetes mellitus (T2DM) postpartum (71), frequently accompanied by more severe insulin resistance and chronic inflammatory states (72). These factors collectively promote tumor growth through activation of the PI3K/Akt/mTOR signaling pathway (71). Third, differences in body composition distribution (obesity): Asian women exhibit higher proportions of visceral adipose tissue. Given that Asian populations have lower BMI cutoff values (73), even within normal BMI ranges, visceral fat accumulation following GDM may exacerbate abnormalities in adipokine (e.g., leptin) secretion, thereby creating a carcinogenic microenvironment (2, 22). Fourth, screening and intervention delays: In some Asian regions, inadequate long-term follow-up of GDM patients fails to effectively manage glucose metabolism disorders, leading to the persistent accumulation of hyperglycemia-related DNA oxidative damage (27). Fifth, breastfeeding practices: Cultural and occupational factors and other socioenvironmental factors in certain Asian regions result in suboptimal breastfeeding practices, preventing the full realization of the lactation-associated reduction in cancer risk, consequently elevating breast cancer incidence (54).

In conclusion, although we have explored the impact of GDM on the development of breast cancer, the existing research results have not elucidated the specific mechanisms of the association between GDM and breast cancer. Therefore, more basic and clinical studies are needed to clarify the relationship between GDM and the risk of breast cancer.

### 4.3 Limitations and advantages

Our meta-analysis has the following limitations:

(1) All included studies were observational and may have been subject to confounding factors and biases. In addition, both the case-control study and the cohort study are observational studies with a relatively low level of evidence; therefore, the quality of evidence derived from our findings is limited.

(2) Differences in the definition and diagnosis codes for GDM among studies could potentially affect the accuracy of the results.

(3) The heterogeneity of the outcome measures was relatively high, and the sources of this heterogeneity were not fully explained.

(4) Due to the limited number of included studies, the subgroup analysis was dominated by studies from China and Korea, leading to insufficient regional representation. Larger sample size studies are needed for further validation in the future.

Despite these limitations, our meta-analysis has several notable strengths:

(1) This study strictly followed the PRISMA guidelines for systematic searching, screening, and data extraction. The process was ensured to be objective through independent double-blind reviews and third-party arbitration. Additionally, the Newcastle-Ottawa Scale was used to assess the quality of the included studies, ensuring high methodological reliability overall.

(2) Subgroup analyses were conducted to explore the effects of region, study design, and follow-up duration. Regional differences were identified as key moderators, providing new directions for future research and comprehensive data integration.

(3) Both Begg’s and Egger’s tests did not reveal significant publication bias, and the funnel plot demonstrated good symmetry, indicating that small sample studies less influenced the results. Sensitivity analysis showed that the main effect estimates were robust and reliable.

(4) This study also explored the potential mechanisms underlying the association between GDM and breast cancer, providing a stronger theoretical foundation for the research conclusions.

### 4.4 Clinical implications

In clinical practice, attention should be paid to the regional differences in the association between GDM and breast cancer risk. Given the observed association in Asian populations with GDM, for Asian patients with GDM, especially those who progress to T2DM or have visceral fat accumulation after childbirth, early screening for breast cancer (such as regular breast ultrasound and mammography) should be strengthened, and the postpartum metabolic follow-up period should be extended. Meanwhile, regardless of the region, lifestyle interventions (such as a low-carbohydrate diet and regular exercise) should be intensified for GDM patients after childbirth to improve insulin resistance and chronic inflammatory status, and breastfeeding should be encouraged to exert its potential protective effects on breast tissue. Future clinical studies can focus on long-term metabolic trajectory monitoring after childbirth in GDM patients and the application of biomarkers in breast cancer risk prediction, providing a scientific basis for individualized prevention strategies.

## 5 Conclusion

Overall, the results of this study indicate that there is no significant association between GDM and the risk of breast cancer. However, significant regional heterogeneity exists: Our findings suggest an association between GDM and reduced breast cancer risk in North American populations, while an association with increased risk was observed in Asian populations. This discrepancy may be related to differences in lifestyle, environmental factors, genetic elements, metabolic characteristics, and medical intervention strategies among regions. Considering the limitations of existing evidence, it is necessary to conduct more large-scale, high-quality clinical studies to clarify the causal Association between GDM and breast cancer and construct a risk prediction model, thus providing a more solid evidential basis for precise clinical prevention.

## Data Availability

The original contributions presented in the study are included in the article/**Supplementary Material**. Further inquiries can be directed to the corresponding author.
