# Supplementary material for: Association between gestational diabetes mellitus and risk of breast cancer: a systematic review and meta-analysis
Source: Front Endocrinol (Lausanne). 2025 Jul 3;16:1621932. doi: 10.3389/fendo.2025.1621932 (PMC12267041; doi:10.3389/fendo.2025.1621932)

**Association Between Gestational Diabetes Mellitus and Risk of Breast Cancer: A Systematic Review and Meta-Analysis**

[1 Table S1 Newcastle-Ottawa Quality Assessment Scale - Quality Assessment of Included Cohort Study. 2](#_Toc17307)

[2 Table S2 Newcastle-Ottawa Quality Assessment Scale - Quality Assessment of Included Case-control Study. 4](#_Toc3792)

[3 Figure Legends: 5](#_Toc7895)

[4 Figure S1: Begg's test and Egger's test 5](#_Toc8065)

# 1 Table S1 Newcastle-Ottawa Quality Assessment Scale - Quality Assessment of Included Cohort Study.

| Author (year) | Selection (Max 4 points) | | | | Comparability (Max 2 points) | Outcome | | | Total Score (Max 9 points) |
| --- | --- | --- | --- | --- | --- | --- | --- | --- | --- |
|  | Representativeness of the Exposed Cohort | Selection of the Non-Exposed Cohort | Ascertainment of Exposure | Demonstration That Outcome of Interest Was Not Present at Start of Study | Comparability of Cohorts on the Basis of the Design or Analysis | Assessment of Outcome | Was Follow-Up Long Enough for Outcomes to Occur | Adequacy of Follow Up of Cohorts |  |
| Gurjot Gill MD,2024 | 1 | 1 | 1 | 0 | 2 | 1 | 1 | 0 | High (7) |
| Kimberly A Bertrand,2021 | 1 | 1 | 1 | 0 | 2 | 1 | 1 | 0 | High (7) |
| Tal Sella,2011 | 1 | 1 | 1 | 0 | 2 | 1 | 1 | 1 | High (8) |
| Oded Fuchs,2017 | 1 | 1 | 1 | 0 | 2 | 1 | 1 | 0 | High (7) |
| Kyu-Tae Han,2018 | 1 | 1 | 1 | 0 | 2 | 1 | 1 | 1 | High (8) |
| YunShing Peng,2019 | 1 | 1 | 1 | 0 | 2 | 1 | 1 | 0 | High (7) |
| Yong-Moon Mark Park,2017 | 1 | 1 | 1 | 0 | 2 | 1 | 1 | 1 | High (8) |
| S.A.D.Bejaimal,2015 | 1 | 1 | 1 | 0 | 2 | 1 | 1 | 0 | High (7) |
| Kimberly A. Bertrand,2020 | 1 | 1 | 1 | 0 | 2 | 1 | 1 | 1 | High (8) |
| Camille E. Powe,2017 | 1 | 1 | 1 | 0 | 2 | 1 | 1 |  | High (7) |
| Romina Pace,2020 | 1 | 1 | 1 | 0 | 2 | 1 | 1 |  | High (7) |
| M. C. Perrin,2008 | 1 | 1 | 1 | 0 | 2 | 1 | 1 | 1 | High (8) |
| Maria Hornstrup Christensen,2024 | 1 | 1 | 1 | 0 | 2 | 1 | 1 |  | High (7) |
| Sungmin Park，2022 | 1 | 1 | 1 | 0 | 2 | 1 | 1 |  | High (7) |

# 2 Table S2 Newcastle-Ottawa Quality Assessment Scale - Quality Assessment of Included Case-control Study.

| Author (year) | Selection (Max 4 points) | | | | Comparability (Max 2 points) | Outcome | | | Total Score (Max 9 points) |
| --- | --- | --- | --- | --- | --- | --- | --- | --- | --- |
|  | Is the Case Definition Adequate | Representativeness of the Cases | Selection of Controls | Definition of Controls | Comparability of Cases and Controls on the Basis of the Design or Analysis | Ascertainment of Exposure | Same Ascertainment Method | Non-Response Rate |  |
| Theodore M. Brasky,2013 | 1 | 1 | 0 | 1 | 2 | 1 | 1 | 0 | High (7) |
| Arash Ardalan,2016 | 1 | 1 | 1 | 1 | 2 | 1 | 1 | 0 | High (8) |
| Maureen Sanderson,2010 | 1 | 1 | 0 | 1 | 2 | 1 | 1 | 0 | High (7) |
| Dana E. Rollison,2008 | 1 | 1 | 1 | 1 | 2 | 1 | 1 | 0 | High (8) |
| Rebecca Troisi,1998 | 1 | 1 | 0 | 1 | 1 | 1 | 0 | 0 | Medium (5) |

# 3 Figure Legends:

**Figure 1**: PRISMA flow chart for study selection.

**Figure 2**: Forest plot of the association between GDM and breast cancer.

**Figure 3**: Subgroup analysis - region.

**Figure 4**: Subgroup analysis - study design.

**Figure 5**: Subgroup analysis - follow-up duration.

**Figure 6**: Sensitivity analyses.

**Figure 7**: Funnel plot.

# 4 Figure S1: Begg's test and Egger's test


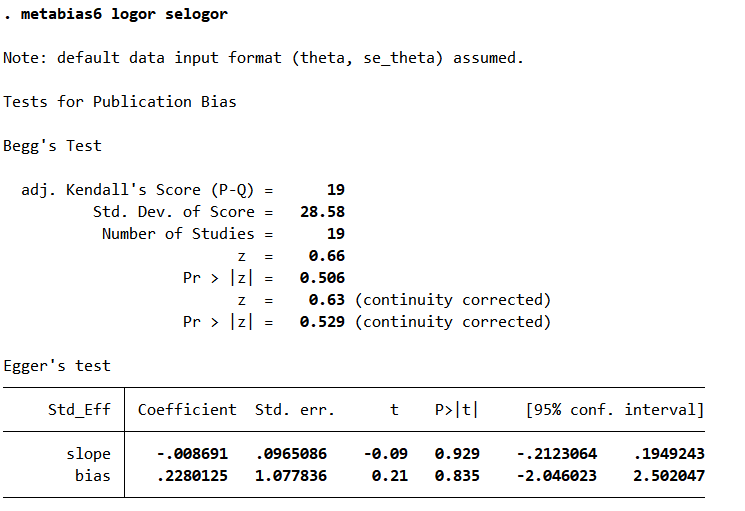

Supplement: Supplementary file 1 [file DataSheet1.docx]
